# Identification of a Sixteen-gene Prognostic Biomarker for Lung Adenocarcinoma Using a Machine Learning Method

**DOI:** 10.7150/jca.34585

**Published:** 2020-01-01

**Authors:** Baoshan Ma, Yao Geng, Fanyu Meng, Ge Yan, Fengju Song

**Affiliations:** 1 College of Information Science and Technology, Dalian Maritime University, Dalian 116026, China;; 2 Department of Epidemiology and Biostatistics, Key Laboratory of Cancer Prevention and Therapy, Tianjin, National Clinical Research Center of Cancer, Tianjin Medical University Cancer Institute and Hospital, Tianjin 300060, China.

**Keywords:** Lung adenocarcinoma, Prognosis prediction, RNA-Seq data, Random survival forest, Forward selection model

## Abstract

**Objectives**: Lung adenocarcinoma (LUAD) accounts for a majority of cancer-related deaths worldwide annually. The identification of prognostic biomarkers and prediction of prognosis for LUAD patients is necessary.

**Materials and Methods**: In this study, LUAD RNA-Seq data and clinical data from the Cancer Genome Atlas (TCGA) were divided into TCGA cohort I (n = 338) and II (n = 168). The cohort I was used for model construction, and the cohort II and data from Gene Expression Omnibus (GSE72094 cohort, n = 393; GSE11969 cohort, n = 149) were utilized for validation. First, the survival-related seed genes were selected from the cohort I using the machine learning model (random survival forest, RSF), and then in order to improve prediction accuracy, the forward selection model was utilized to identify the prognosis-related key genes among the seed genes using the clinically-integrated RNA-Seq data. Second, the survival risk score system was constructed by using these key genes in the cohort II, the GSE72094 cohort and the GSE11969 cohort, and the evaluation metrics such as HR, *p* value and C-index were calculated to validate the proposed method. Third, the developed approach was compared with the previous five prediction models. Finally, bioinformatics analyses (pathway, heatmap, protein-gene interaction network) have been applied to the identified seed genes and key genes.

**Results and Conclusion**: Based on the RSF model and clinically-integrated RNA-Seq data, we identified sixteen key genes that formed the prognostic gene expression signature. These sixteen key genes could achieve a strong power for prognostic prediction of LUAD patients in cohort II (HR = 3.80, *p* = 1.63e-06, C-index = 0.656), and were further validated in the GSE72094 cohort (HR = 4.12, *p* = 1.34e-10, C-index = 0.672) and GSE11969 cohort (HR = 3.87, *p* = 6.81e-07, C-index = 0.670). The experimental results of three independent validation cohorts showed that compared with the traditional Cox model and the use of standalone RNA-Seq data, the machine-learning-based method effectively improved the prediction accuracy of LUAD prognosis, and the derived model was also superior to the other five existing prediction models. KEGG pathway analysis found eleven of the sixteen genes were associated with Nicotine addiction. Thirteen of the sixteen genes were reported for the first time as the LUAD prognosis-related key genes. In conclusion, we developed a sixteen-gene prognostic marker for LUAD, which may provide a powerful prognostic tool for precision oncology.

## Introduction

Lung cancer is the leading cause of cancer death worldwide, and approximately 1 million people die of this disease each year 1-3. Lung adenocarcinoma (LUAD) is the most common histological subtype of lung cancer, and the incidence and mortality are increasing 4-6. The average 5-year survival rate for LUAD patients is only 15%, although progress has been made in treatment, such as combination of chemotherapy and chemoradiation, survival rate has been improved very little over the past a few decades 7-9. Therefore, it is particularly important to find prognostic markers for LUAD and provide precise targeted therapy.

With the development of high-throughput sequencing techniques to interrogate genome-wide genetic variations, RNA-Seq based markers have recently been studied as prognostic markers for lung cancer 10, 11. Sudbanshu Shukla et al. undertook the first prognostic analysis of LUAD RNA-Seq data and generated the prognostic feature using the Cox model, which can provide a powerful prognostic tool for precision oncology as part of an integrated RNA-Seq clinical sequencing program 5. Subsequently, Li B et al. used the RNA-Seq dataset to develop a robust, individualized clinical immune signature that can assess the prognosis and overall survival in patients with early-stage nonsquamous non-small cell lung cancer 12. As a subset of RNA-Seq, certain long non-coding RNAs (lncRNAs) also encode proteins and play crucial roles in gene transcription and regulation 13, 14. Zheng S et al. used the Cox model to develop an 8-lncRNA prognostic signature in LUAD patients, which provided an effective independent prognostic prediction model for LUAD patients 15. These studies were based solely on RNA-Seq data. Yuan Yuan et al. used molecular data in combination with clinical variables to predict the survival of four cancer types, the results showed that the comprehensive model had little predictive power for lung squamous cell carcinoma (LUSC), while the predictive power of the other three kinds of cancers (kidney renal clear cell carcinoma, glioblastoma multiforme, ovarian serous cystadenocarcinoma) were significantly improved 16.

Broadly defined, machine learning is a branch of computer science that deals with making predictions from complex data through statistical models and is widely applied in the biomedical field 17, 18. As an efficient machine learning algorithm, RSF is considered as a more powerful method for survival analysis 19, 20. In the present study, the high-throughput RNA-Seq data and clinical data were downloaded from the TCGA database, the Cox model and the RSF model were used to identify the survival-related seed genes from the TCGA cohort I, and then the forward selection model was generated and the prognosis-related key genes were identified. Finally, based on the RSF model and the clinically-integrated RNA-Seq data, we obtained a subset of sixteen genes that formed the prognostic gene expression signature and improved the power for predicting the prognosis of LUAD patients.

## Materials and Methods

### Data collection and preprocessing

The RNA-Seq data and the corresponding clinical information for LUAD were downloaded from the publicly available TCGA database. After filtering out the missing data, a total of 506 LUAD patients were kept as our study samples. The 506 LUAD patients were further randomly assigned to a training cohort (TCGA cohort I, n = 338) and an internal validation cohort (TCGA cohort II, n = 168). Moreover, the other two external validation cohorts consisting of 393 and 149 LUAD patients were downloaded from the GEO database (GSE72094 cohort, GSE11969 cohort). In the RNA-Seq dataset for the above four cohorts, the numerical distribution of Reads per Kilo-base per Million mapped (RPKM) reads are too wide to be used in model analysis, thus we formulated each RPKM value in 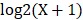, where *X* is the RPKM value 21.

### Machine learning model: random survival forest

Random survival forest (RSF) is an adaptation of random forests (RF) designed to be used for survival data 22. The mathematical principle of the RSF model is introduced as follows: bootstrap methods are used to randomly extract the *ntree* bootstrap samples from the raw data and create a binary recursive survival tree for each sample. In the experiment, the good division was determined by the log-rank splitting rule to maximize the survival difference between the daughter nodes 23. The Cumulative Hazard Function (CHF) gives the values of the terminal nodes associated with time. For a terminal node *h* of a survival tree *n* at time *t*, this is given by the Nelson-Aalen estimator:


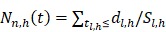


Among them, 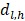 represents the number of deaths, 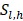 indicates individuals at risk, 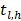 indicates distinct time events. All cases of 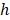 are assigned the same CHF. In order to calculate the ensemble CHF of the survival forest of *ntree* trees with a given d-dimensional case 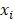,


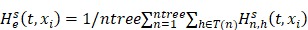


Where 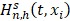 is equal to the Nelson-Aalen estimator, if 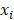 ends with the survival tree falling to *h* 24,


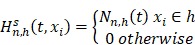


The RSF model provides a unified treatment of Breiman's RF 25 for a variety of data settings, such as survival, regression, classification and so on. Survival is grown for right-censored survival data. Survival settings require a time and censoring variable which should be identified as the response in the *Surv* function for the “randomForestSRC” R package (http://www.r-project.org) 26. The censoring variable must be coded as a non-negative integer with 0 reserved for censor and 1 reserved for death. Furthermore, this model generates a random forest using the training data, and the function *var.select* implements random forest variable selection using tree minimal depth methodology 25, 27.

### Survival-related seed genes generation

The association between genes and patients' overall survival was analyzed in the TCGA cohort I. The survival-related seed genes were screened and identified from all the genes using the following two models.

All the genes among the TCGA cohort I were included in the Cox univariate survival analysis by the “survival” R package, the genes with expressing significance *p* values less than 0.05 were extracted as the first group of survival-related seed genes. The Cox model is a traditional method in the biostatistical field. In addition, the RSF model in machine learning field was used to select the second group of survival-related seed genes from all the genes among the TCGA cohort I again, which was built by the “randomForestSRC” R package 26.

To search the key pathways that were associated with LUAD survival, we next performed the KEGG pathway enrichment analysis for the two sets of survival-related seed genes by using the Database for Annotation, Visualization and Integrated Discovery (DAVID) 28, 29.

### Prognosis-related key genes selection

The seed genes in primary selection were not suitable for clinical diagnosis 21. To increase the feasibility and reliability of clinical prognosis based on gene, the forward selection model was used to select the prognosis-related key genes from seed genes. We implemented prognosis-related key genes selection by the “rbsurv” R package 30-32. The procedure was as followings:

(I) All the samples of the TCGA cohort I were randomly divided into 2/3 training set and 1/3 validation set. A gene was fitted into the training set and the parameter estimate 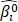 of this gene was obtained. Then the parameter estimate 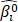 and the validation set were used to calculate the log-likelihood. This evaluation was carried out for each gene.

(II) The above procedure was repeated *n* times, and each gene obtained *n* log-likelihoods. The gene with the largest mean log-likelihood was selected as the top gene and labeled g (1). Subsequently, we searched the next top two genes by evaluating every two-gene model and selected an optimal one with the largest mean log-likelihood.

(III) We continued this forward gene selection procedure and eventually generated a series of models. In order to prevent overfitting, the akaike information criterion (AIC) was used to evaluate these modes, rather than log-likelihood. Finally, the best model with minimum AIC was selected.

(IV) Risk factors were included in the model. Then steps II-IV were repeated.

### Verification and comparison of key genes

In order to comprehensively investigate the association between these key genes and the prognosis of LUAD, we developed the survival risk score systems using the TCGA cohort II, the GSE72094 cohort and the GSE11969 cohort. The risk scores were calculated by taking into account both the expression data of these prognosis-related key genes and correlation coefficients. Then the patients of the TCGA cohort II, the GSE72094 cohort and the GSE11969 cohort were divided into two groups separately at high- and low-risk using the 50th percentile of risk score 33. The KM survival curves were used to assess the efficacy of key genes in the three groups. We then calculated the concordance index (C-index) using function *rcorr.cens* in the R “Hmisc” package 34. In addition, previous studies used various methods (such as decision trees, risk scoring, semi-supervised, and various online tools) to obtain prognostic genetic features of lung cancer or non-small cell lung cancer 5, 35-38. We compared the sixteen-gene prognostic signatures to the five published lung cancer prognostic signatures by rederiving a multivariable Cox model using the gene list from each signature.

## Results

**Figure 1:**
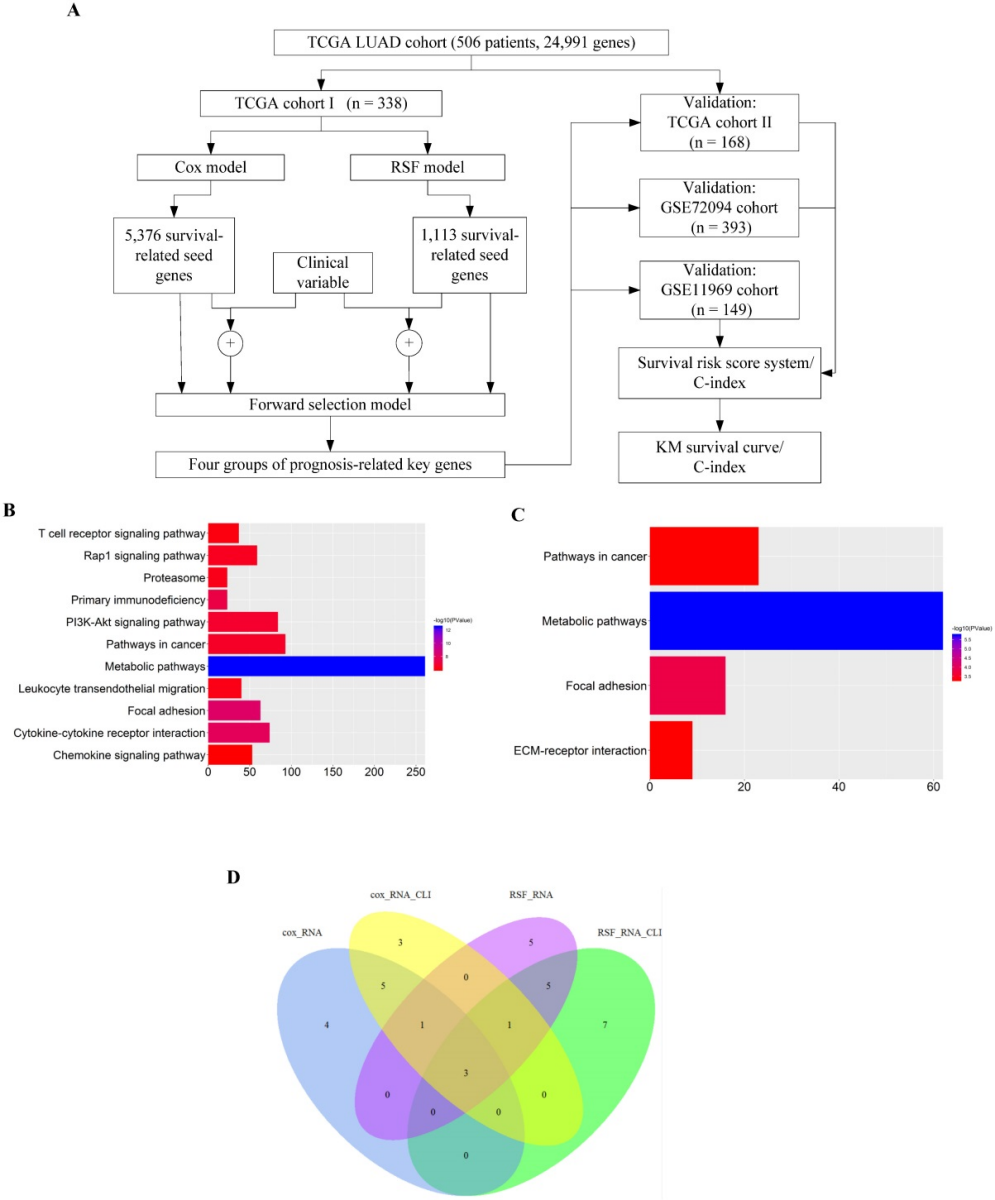
Identification of prognostic gene signature. **A)** Flowchart of RNA-Seq analysis and signature generation. Briefly, survival-related seed genes of the 506 TCGA LUAD patients were first identified by the Cox model and the machine learning model (random survival forest, RSF) from the TCGA cohort I. Next the forward selection model was used to select four sets of key genes for prognosis prediction. The survival risk score systems were built based on the expression data of gene signatures in the TCGA cohort II and the GSE72094 cohort and the GSE11969 cohort, which divided patients into high- and low-risk groups. **B)** KEGG enrichment pathway analysis of 5376 survival-related seed genes obtained by the Cox model. **C)** KEGG enrichment pathway analysis of 1113 survival-related seed genes obtained by the RSF model. **D)** The venn diagram showed that the common key genes obtained from RNA-Seq data and clinically-integrated RNA-Seq data using both the Cox model and the RSF model.

The overall flowchart of this work was summarized in Fig. 1A. Firstly, the Cox model and the RSF model were used to screen each one group of survival-related seed genes from all the genes in the TCGA cohort I, and then the forward selection model employed the seed genes or the clinically-integrated seed genes to select four sets of prognosis-related key genes. Finally, the TCGA cohort II, the GSE72094 cohort and the GSE11969 cohort were used to verify the performance of the four sets of prognosis-related key genes.

### Identification of seed genes associated with survival

The full TCGA cohort included 24991 genes and 506 samples, each sample had survival time and survival status. The Cox univariate survival analysis model and the RSF model were used respectively to select the survival-related seed genes from all the 24 991 genes in TCGA cohort I. The Cox model showed that 5376 genes were statistically significantly correlated with overall survival at the* p* value less than 0.05. Besides, in the RSF model, 1113 genes were screened using the minimum depth selection method. Pathway analysis showed that the 5376 genes were statistically significantly enriched for Metabolic pathways (*p* = 3.28e-13) and Focal adhesion (*p* = 6.14e-09) (Fig. 1B), and the 1113 genes were statistically significantly enriched for Metabolic pathways (*p* = 1.85e-06) (Fig. 1C). These two sets of survival-related seed genes were used for further analysis.

### Identification of key genes associated with prognosis

We selected the prognosis-related key genes from the two groups of seed genes based on the forward selection model. We firstly performed the Cox survival analysis for clinical data of the TCGA cohort I, and selected the risk factors with *p* value less than 0.05 for construction of clinically-integrated RNA-Seq data (Supplementary Table S1). In the TCGA cohort I, a series of gene models were generated, and then the best model was selected by the minimum AIC. The best model identified by the Cox model and RNA-Seq data, the Cox model and clinically-integrated RNA-Seq data, the RSF model and RNA-Seq data, the RSF model and clinically-integrated RNA-Seq data respectively contained 13, 13, 15 and 16 genes (Supplementary Table S2, S3, S4 and S5). In addition, the parameters in the forward selection model were shown in Supplementary Table S6. The venn diagram showed that four genes were both selected from the Cox model and the RSF model (Fig. 1D).

### Development of survival risk score system

**Figure 2:**
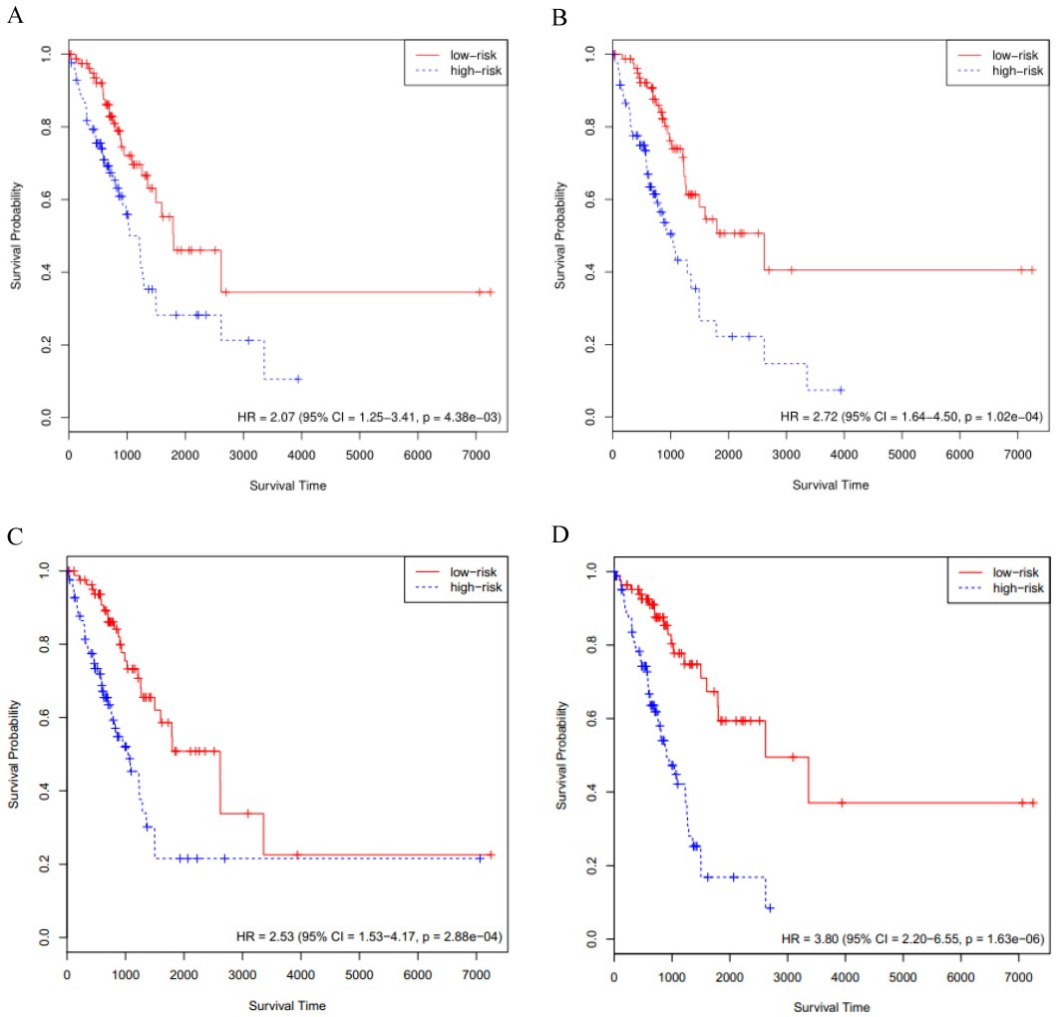
Validation of the prognosis-related key genes in the TCGA cohort II. **A)** The KM survival curve was generated in the TCGA cohort II by the Cox model and RNA-Seq data. Patients of the TCGA cohort II were divided into high- and low-risk groups based on the 50th percentile of risk score. **B)** The KM survival curve was generated in the TCGA cohort II by the Cox model and clinically-integrated RNA-Seq data. Patients of the TCGA cohort II were divided into high- and low-risk groups based on the 50th percentile of risk score. **C)** The KM survival curve was generated in the TCGA cohort II by the RSF model and RNA-Seq data. Patients of the TCGA cohort II were divided into high- and low-risk groups based on the 50th percentile of risk score. **D)** The KM survival curve was generated in the TCGA cohort II by the RSF model and clinically-integrated RNA-Seq data. Patients of the TCGA cohort II were divided into high- and low-risk groups based on the 50th percentile of risk score.

We employed the four sets of prognosis-related key genes to construct four survival risk score systems in the TCGA cohort II. The risk scores were calculated by taking into account the expression data of these prognosis-related key genes and correlation coefficients (Supplementary Table S2, S3, S4 and S5), and a threshold was set at the 50th percentile. The higher the risk score, the larger mortality risk for LUAD patients. Then the patients of the TCGA cohort II were divided into high- and low-risk groups based on the threshold. High-risk patients, as defined by the four groups of prognosis-related key genes based on the risk score, had statistically significantly worse prognosis in TCGA cohort II (Fig. 2A, B, C and D). Then through the assessment of the four survival risk score systems (Table 1), the RSF model showed higher predictive power than the Cox model, and the predictive power of clinically-integrated RNA-Seq data was higher than RNA-Seq data. In short, the RSF model and clinically-integrated RNA-Seq data could predict the prognosis of LUAD patients well (HR = 3.80, 95% CI: 2.20-6.55, *p* = 1.63e-06) (Fig. 2D).

#### The C-index

The C-index for the four sets of key genes in the TCGA cohort II was showed in Table 1. The C-index was mainly used to calculate the degree of discrimination between the predicted value of the Cox model and the reality in the survival analysis 39. We calculated the C-index for both the Cox model and the RSF model, the C-index increased for clinically-integrated RNA-Seq data in comparison to the RNA-Seq data. In addition, the C-index of the RSF model was larger than that of the Cox model.

### External validation of key genes in the GSE72094 cohort

#### The KM curves

**Figure 3:**
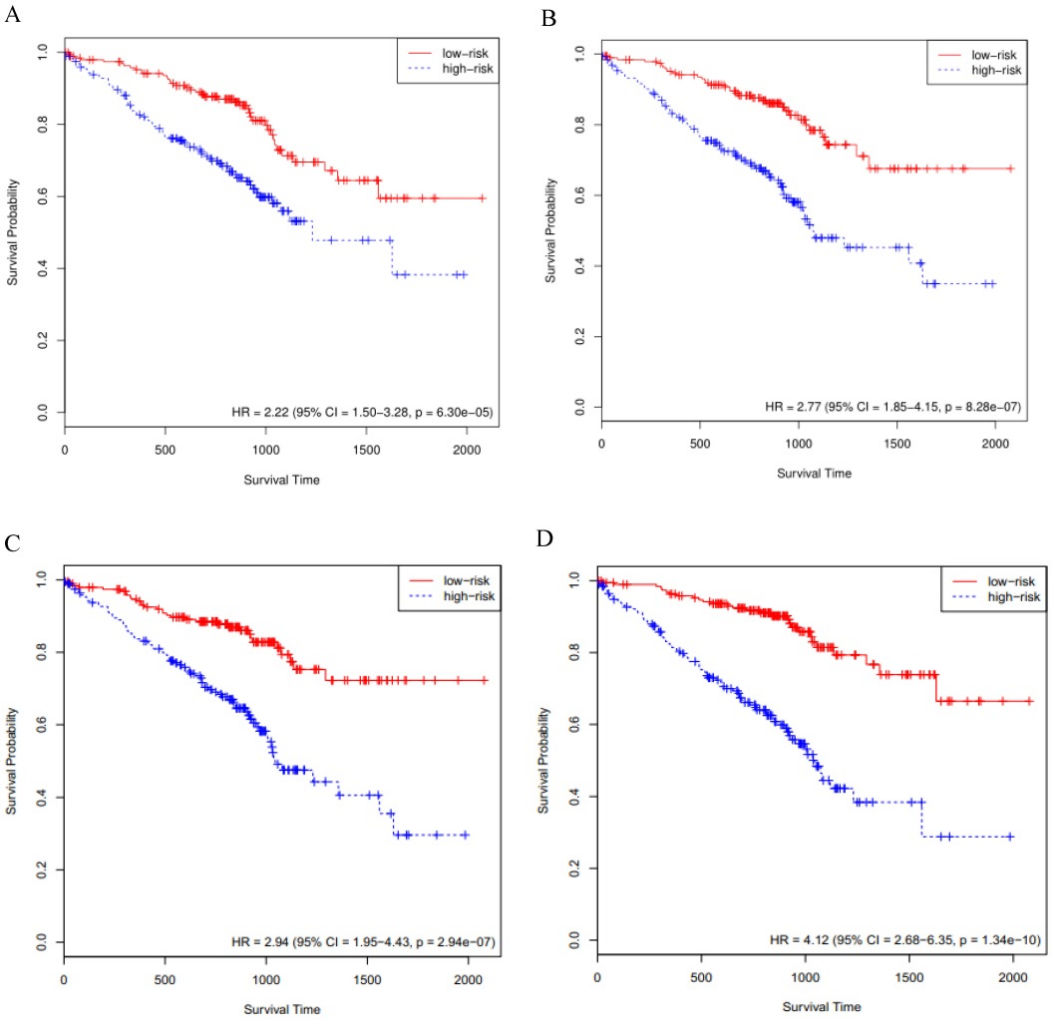
Validation of the prognosis-related key genes in the GSE72094 cohort. **A)** The KM survival curve was generated in the GSE72094 cohort by the Cox model and RNA-Seq data. Patients of the GSE72094 cohort were divided into high- and low-risk groups based on the 50th percentile of risk score. **B)** The KM survival curve was generated in the GSE72094 cohort by the Cox model and clinically-integrated RNA-Seq data. Patients of the GSE72094 cohort were divided into high- and low-risk groups based on the 50th percentile of risk score. **C)** The KM survival curve was generated in the GSE72094 cohort by the RSF model and RNA-Seq data. Patients of the GSE72094 cohort were divided into high- and low-risk groups based on the 50th percentile of risk score. **D)** The KM survival curve was generated in the GSE72094 cohort by the RSF model and clinically-integrated RNA-Seq data**.** Patients of the GSE72094 cohort were divided into high- and low-risk groups based on the 50th percentile of risk score.

The above-mentioned models were further validated by using the GSE72094 cohort data (n=393). We performed the Cox survival analysis for LUAD patients from the GSE72094 cohort (Supplementary Table S7), the *p* value for stage was much less than 0.05. The risk score of each patient was calculated based on the expression data of the four sets of prognosis-related key genes. We divided 393 LUAD patients of the GSE72094 cohort into high- and low-risk groups, based on the 50th percentile of risk score. The KM curves of the four sets of prognosis-related key genes all indicated that high-risk patients had statistically significantly worse prognosis (Fig. 3A, B, C and D). Furthermore, we compared the four KM survival curves obtained by the four groups of key genes (Table 1), the key genes obtained by the RSF model and clinically-integrated RNA-Seq data can predict the prognosis of LUAD patients well (HR = 4.12, 95% CI: 2.68-6.35, *p* = 1.34e-09) (Fig. 3D).

#### The C-index

The C-index for the four sets of key genes in the GSE72094 cohort was showed in Table 1. We calculated the C-index for both the Cox model and the RSF model, the C-index increased for clinically-integrated RNA-Seq data in comparison to the RNA-Seq data. In addition, the C-index of the RSF model was larger than that of the Cox model. This result is similar to the TCGA cohort II.

### Heat map for the sixteen key genes

**Figure 4:**
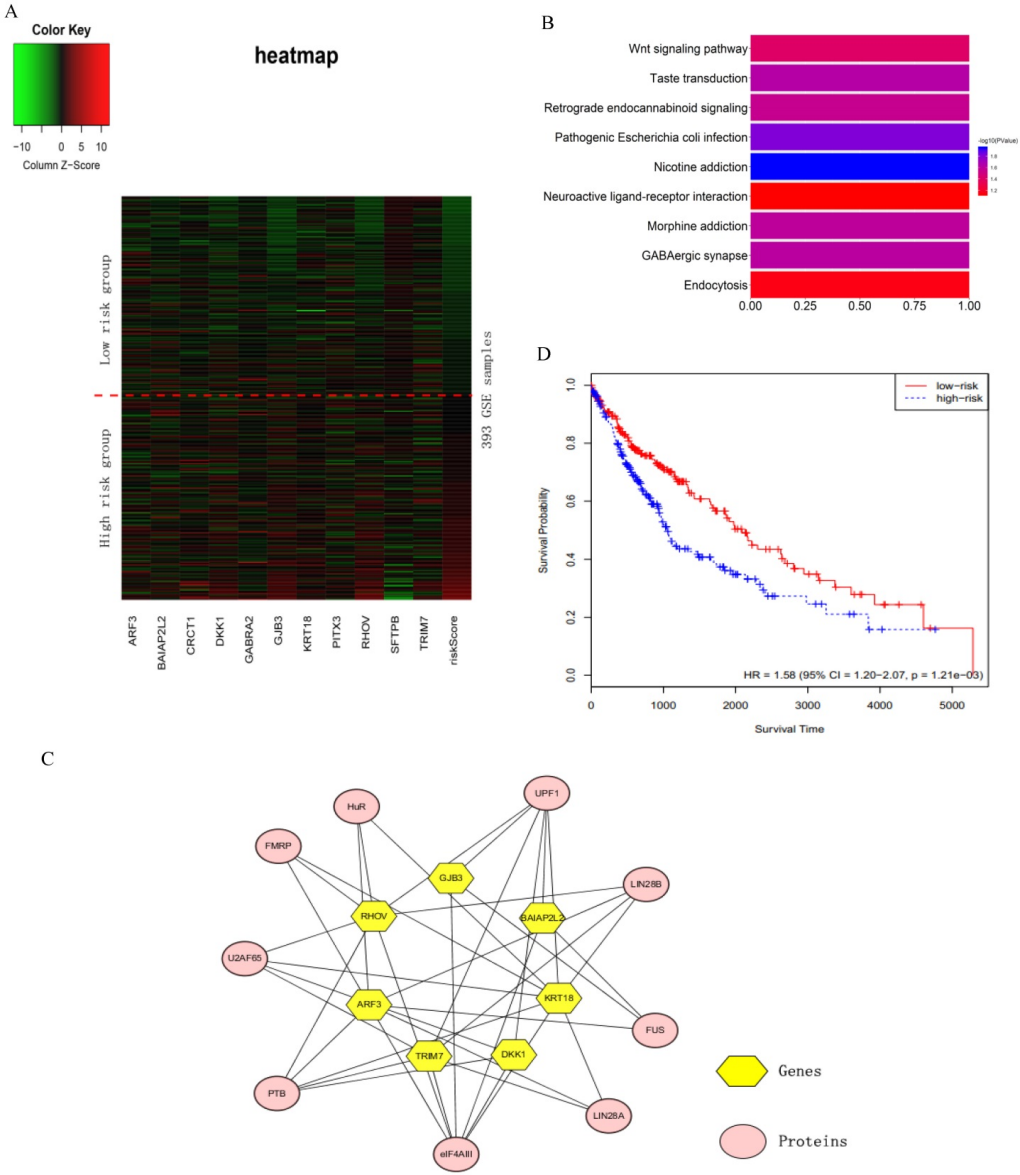
The analysis of the sixteen prognosis-related key genes. **A)** Heat map for the key genes obtained by the RSF model and clinically-integrated RNA-Seq data. The abscissa indicates genes, and the ordinate indicates 393 samples from GSE72094. The rightmost column is the patient's risk score, sorted by ascending order from top to bottom. The low-risk group is above the red dashed line, the high-risk group is under the red dashed line. **B)** KEGG enrichment pathway analysis of the key genes obtained by the RSF model and clinically-integrated RNA-Seq data. **C)** Protein-gene network. The yellow hexagons indicate the genes obtained from the RSF model and clinically-integrated RNA-Seq data, and the orange-red ovals indicate the associated proteins. **D)** The KM survival curve of cross-tumor model.

We verified the sixteen key genes obtained by the RSF model and clinically-integrated RNA-Seq data based on the GSE72094 cohort. Due to the difference of data preprocessing between GEO and TCGA database, GSE72094 cohort only contains eleven common genes among the sixteen key genes. To comprehensively investigate the expression of eleven genes in high- and low-risk groups, we plotted a heat map using the RNA-Seq data of eleven genes, with red color indicating higher expression and green color indicating lower expression (Fig. 4A). The heat map roughly showed that the higher the patient's risk score, the greater the gene expression value. We next performed a KEGG pathway enrichment analysis for the eleven genes by using the DAVID. These eleven genes were statistically significantly enriched for Nicotine addiction (Fig. 4B).

### External validation of key genes in the GSE11969 cohort

These models were further validated by using the GSE11969 cohort data (n = 149). We performed the Cox survival analysis for LUAD patients from the GSE11969 cohort (Supplementary Table S8), the *p* values for stage and age were much less than 0.05. The 149 LUAD patients were divided into high- and low-risk groups by using the same method as the GSE72094 cohort. The KM curves of the four sets of prognosis-related key genes all indicated that high-risk patients had statistically significantly worse prognosis (Supplementary Figure S1A, B, C and D). The C-index for the four sets of key genes in the GSE11969 cohort were showed in the Supplementary Table S9. Furthermore, we compared the four KM survival curves and the C-index derived from the four groups of key genes (Supplementary Table S9), the key genes obtained by the RSF model and clinically-integrated RNA-Seq data can predict the prognosis of LUAD patients well (HR = 3.87, 95% CI: 2.27-6.61, *p* = 6.81e-07) (Supplementary Figure S1D). In addition, by comparing the sixteen genes found in this study with the prognostic features of five published lung cancers (Table 2), we found that sixteen gene prognostic features were statistically significant in the three validation cohorts.

### Interdependency of the sixteen key prognostic genes

The sixteen key genes were LINC00908, PITX3, GJB3, CRCT1, MELTF, BAIAP2L2, RHOV, GABRA2, ARF3, TRIM7, KRT18, ZNF710.AS1, LOC105370802, LOC100996732, SFTPB and DKK1. Proteins interacting with these sixteen key genes were searched in starbase2.0 (http://starbase.sysu.edu.cn/starbase2/index.php). The results were exported as the nodes and edges of a protein-gene network and were visualized by Cytoscape-v3.6.1. In the protein-gene network, the key genes were mainly associated with nine proteins (Fig. 4C).

### Patient prognosis prediction using cross-tumor models

In order to test whether the LUAD patients data could identify commonalities across LUSC, we used the sixteen key gene-trained models obtained from the RSF models and clinically-integrated RNA-Seq data to predict the prognostic characteristics of LUSC patients (n = 486) from the TCGA database. The LUSC patients were divided into high- and low-risk groups according to the 50th percentile of risk score. The KM survival curve showed that the high-risk patients had statistically significantly worse prognosis (HR = 1.58, 95% CI: 1.20-2.07, *p* = 1.21e-03) (Fig. 4D).

## Discussion

Lung cancer is the leading cause of cancer-related mortality, non-small cell lung cancer (NSCLC) accounts for about 80% of the total number of lung cancer patients, while LUAD accounts for more than 40% of NSCLC 40-43. In an effort to bolster clinical tools and biological understanding in LUAD, we presented the RNA-Seq prognostic signatures. We found 1,113 survival-related seed genes from 24,991 genes in the TCGA cohort I using the RSF model, and screened sixteen prognosis-related key genes from the 1,113 clinically-integrated seed genes in TCGA cohort I using the forward selection model. Subsequently the sixteen gene signatures were validated in the TCGA cohort II and the GSE72094 cohort. The sixteen gene signatures can improve the power for predicting the prognosis of LUAD patients.

Previous studies had also found genes associated with lung cancer using methods such as decision trees and Cox models. The sixteen gene prognostic markers obtained in this study had the highest HR and C-index and the lowest *p* of likelihood ratio compared to the prognostic genes identified from these existing studies. This further validated the effectiveness of using the RSF model and clinically-integrated RNA-Seq data to predict prognosis for LUAD patients.

This study proposed a method for predicting LUAD prognosis markers based on machine learning. The RSF is computed from a set of binary decision trees and can be used to select the most important variables that are linked with time to event 44. The Cox model is a traditional regression model which is not based on any assumptions about the nature or shape of the underlying survival distribution 45, 46. The advantage of RSF is that it is more suitable for the automation of survival analysis than the Cox model, because it requires less input from the user in the highly relevant data settings for covariates 45. In addition, by means of the forward selection model, we can identify multiple sets of genes rather than one large set of genes while adjusting for risk factors.

The expressions of the sixteen key genes were closely associated with the LUAD prognosis. Pubmed was searched for articles on these sixteen key genes related to LUAD. Up to Nov 16, 2018, except for RHOV, SFTPB and DKK1, all other genes among the sixteen key genes were the first time to be identified in LUAD samples. In previous study, RHOV is an atypical RHO GTPase that has been nominated as upregulated in non-small cell lung cancer in a minor study 47. Pro-SFTPB is over expressed in non-small cell lung cancer, especially in LUAD 48. In a multivariate analysis of patients with LUAD, DKK1 was independently associated with poor survival 49. This result may play a guiding role in prognosis prediction and targeted therapy in patients with LUAD.

The RNA-Seq data for individualized therapy intensification has the advantages of high throughput, high sensitivity and high efficiency 12, 50. In addition, the sixteen gene signatures may provide clues for targeted therapy. However, this study also had some limitations. Due to data limitation of the public databases, we only considered three clinical variables (age, gender and tumor stage) in the forward selection model, and future studies may include more clinical variables into the model. The derived prognostic model also needs be validated using more independent cohorts and biological experiments.

In this study, first of all, a machine-learning-based method (RSF) is proposed to identify the seed genes for LUAD prognosis. Second, the clinical data (such as stage, age) are integrated with RNA-Seq data to improve prediction accuracy. Third, three independent cohorts (the TCGA cohort II, GSE72094 cohort and GSE11969 cohort) are utilized to validate the proposed method and the results show that the derived sixteen-gene signature is also superior to the previous five prediction models. Fourth, bioinformatics analyses have been used to the identified seed genes and key genes, and thirteen of the sixteen genes are reported as the key genes related to LUAD prognosis for the first time, which could contribute to the new findings of our study distinct with the previous studies. Finally, we also discussed some limitations of this study. To the best of our knowledge, the average 5-year survival rate for LUAD patients is only 15%, and the sixteen gene markers found in this study are especially vital for improving the prognosis prediction of LUAD patients. In the future, we could consider the application of advanced machine learning methods, such as deep learning for the prognostic prediction of cancer, and provide more powerful tools for improving targeted therapy.

## Supplementary Material

Supplementary figure and tables.

## Figures and Tables

**Table 1:** Survival analysis based on the TCGA cohort II and GSE72094 cohort

	TCGA cohort II			GSE72094 cohort		
model	HR^a^ (95% CI^b^)	*p*	C-index^c^	HR (95% CI)	*p*	C-index
model 1^d^	2.07 (1.25-3.41)	4.38e-03	0.610	2.22 (1.50-3.28)	6.30e-05	0.615
model 2^e^	2.72 (1.64-4.50)	1.02e-04	0.645	2.77 (1.85-4.15)	8.28e-07	0.630
model 3^f^	2.53 (1.53-4.17)	2.88e-04	0.643	2.94 (1.95-4.43)	2.94e-07	0.623
model 4^g^	3.80 (2.20-6.55)	1.63e-06	0.656	4.12 (2.68-6.35)	1.34e-10	0.672

^a^HR = hazard ratio; ^b^CI = confidence interval; ^c^C-index = concordance index; ^d^model 1: the Cox model and RNA-Seq data; ^e^model 2: the Cox model and clinically-integrated RNA-Seq data; ^f^model 3: the RSF model and RNA-Seq data; ^g^model 4: the RSF model and clinically-integrated RNA-Seq data.

**Table 2:** Comparison of the sixteen-gene prognostic signatures to the five published lung cancer prognostic signatures

studies	TCGA cohort II			GSE72094 cohort			GSE11969 cohort		
	HR (95% CI)	*p*	C-index	HR (95% CI)	*p*	C-index	HR (95% CI)	*p*	C-index
Present study 16-gene signature	3.80 (2.20-6.55)	1.63e-06	0.656	4.12 (2.68-6.35)	1.34e-10	0.672	3.87 (2.27-6.61)	6.81e-07	0.670
Shukla et al. 4-gene signature	2.24 (1.36-3.68)	1.48e-03	0.613	3.01 (2.00-4.51)	1.07e-07	0.639	3.02 (1.85-4.92)	9.42e-06	0.641
Boutros et al. 6-gene signature	1.70 (1.04-2.77)	3.49e-02	0.561	3.17 (2.10-4.79)	3.84e-08	0.656	3.09 (1.89-5.03)	6.10e-06	0.646
Chen et al. 5-gene signature	2.20 (1.34-3.61)	1.93e-03	0.625	2.83 (1.88-4.27)	7.07e-07	0.631	3.09 (1.89-5.03)	6.27e-06	0.644
Lau et al. 3-gene signature	1.80 (1.10-2.94)	1.88e-02	0.588	2.81 (1.88-4.22)	5.55e-07	0.625	2.62 (1.62-4.23)	7.90e-05	0.628
Bianchi et al. 10-gene signature	3.13 (1.84-5.34)	2.78e-05	0.619	2.92 (1.94-4.39)	2.53e-07	0.640	3.41 (2.08-5.59)	1.23e-06	0.655

## Abbreviations

AIC: akaike information criterion
CHF: Cumulative Hazard Function
C-index: concordance index
DAVID: Database for Annotation, Visualization and Integrated Discovery
lncRNAs: long non-coding RNAs
LUAD: Lung adenocarcinoma
LUSC: lung squamous cell carcinoma
NSCLC: non-small cell lung cancer
RF: random forests
RPKM: Reads per Kilo-base per Million mapped
RSF: random survival forest
TCGA: the Cancer Genome Atlas
