# Supplementary material for: Identification of a Sixteen-gene Prognostic Biomarker for Lung Adenocarcinoma Using a Machine Learning Method
Source: J Cancer. 2020 Jan 1;11(5):1288–98. doi: 10.7150/jca.34585 (PMC6959071; doi:10.7150/jca.34585)
Supplement: Supplementary file 1 — Supplementary figure and tables. [file jcav11p1288s1.pdf]

Figure Legend

Figure S1: Validation of the prognosis-related key genes in the GSE11969 cohort. **A)** The KM survival curve was generated in the GSE11969 cohort by the Cox model and RNA-Seq data. Patients of the GSE11969 cohort were divided into high- and low-risk groups based on the 50th percentile of risk score. **B)** The KM survival curve was generated in the GSE11969 cohort by the Cox model and clinically-integrated RNA-Seq data. Patients of the GSE11969 cohort were divided into high- and low-risk groups based on the 50th percentile of risk score. **C)** The KM survival curve was generated in the GSE11969 cohort by the RSF model and RNA-Seq data. Patients of the GSE11969 cohort were divided into high- and low-risk groups based on the 50th percentile of risk score. **D)** The KM survival curve was generated in the GSE11969 cohort by the RSF model and clinically-integrated RNA-Seq data**.** Patients of the GSE11969 cohort were divided into high- and low-risk groups based on the 50th percentile of risk score.

Figure S1

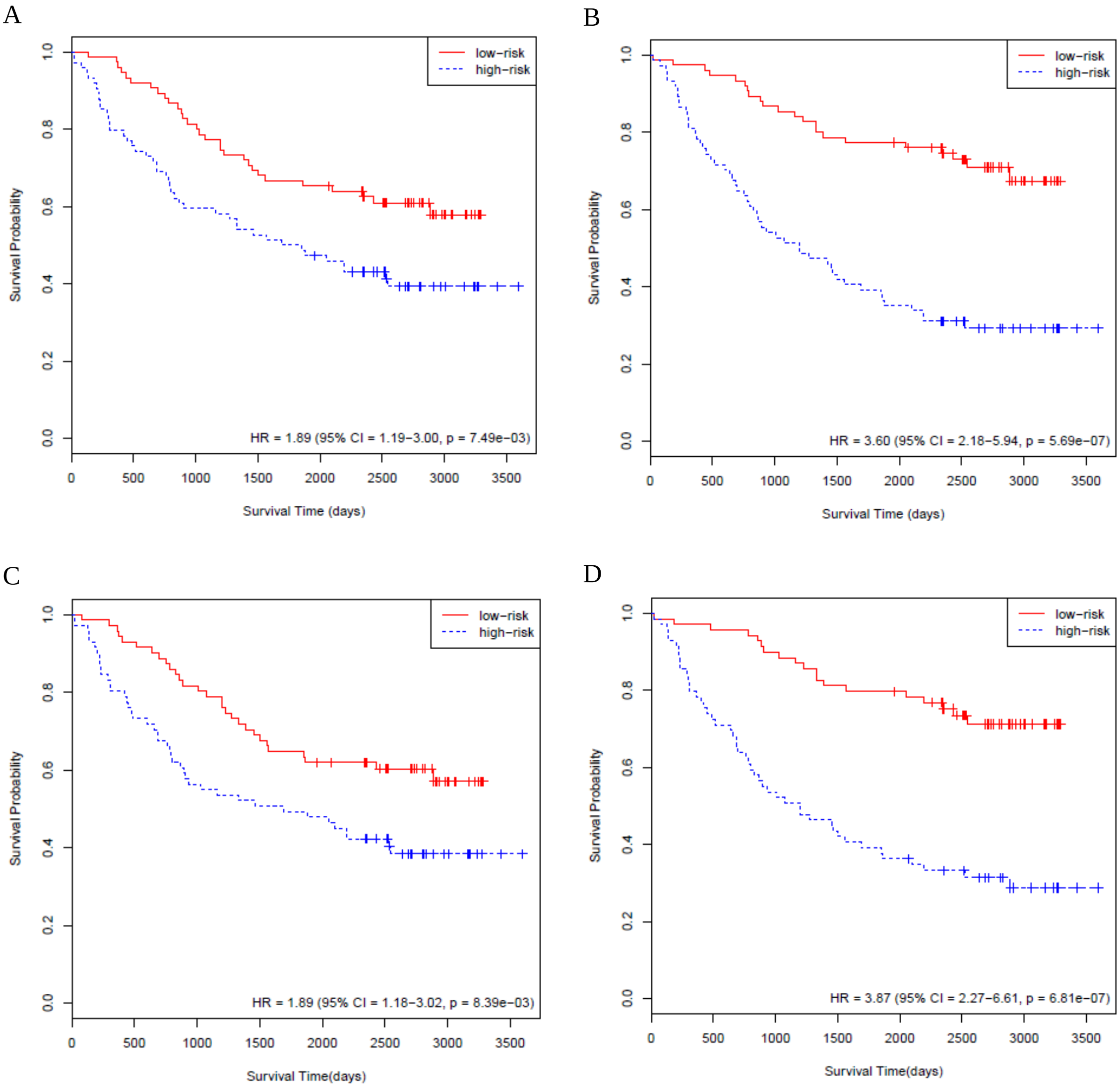
A
low−risk
high−risk
Survival Probability
Survival Time (days)
HR = 1.89 (95% CI = 1.19−3.00, p = 7.49e−03)
B
low−risk
high−risk
Survival Probability
Survival Time (days)
HR = 3.60 (95% CI = 2.18−5.94, p = 5.69e−07)
C
low−risk
high−risk
Survival Probability
Survival Time(days)
HR = 1.89 (95% CI = 1.18−3.02, p = 8.39e−03)
D
low−risk
high−risk
Survival Probability
Survival Time(days)
HR = 3.87 (95% CI = 2.27−6.61, p = 6.81e−07)

Table S1: Cox survival analysis of the TCGA cohort I

Factor	Univariate		Multivariable	
	HR[a](95% CI[b])	*p*	HR(95% CI)	*p*
age(>65.5[c] vs <=65.5)	1.21 (0.84 to 1.73)	0.31	1.19 (0.82 to 1.73)	0.37
gender(female vs male)	0.72 (0.50 to 1.03)	0.07	0.90 (0.62 to 1.32)	0.60
stage	1.78 (1.50 to 2.11)	3.95e-11	1.48 (1.15 to 1.90)	2.23e-03
risk score (high vs low)	3.14 (2.11 to 4.69)	2.04e-08	1.74 (0.99 to 3.08)	0.06

[a]HR = hazard ratio;
[b]CI = confidence interval;
[c]65.5 = median age of patients with LUAD in the TCGA cohort I.

Table S2: Prognosis-related key genes screening by the Cox model and RNA-Seq data

nloglik	AIC	gene symbol	regression coefficients
567.03	1136.07*	LINC00683	-0.14113
557.79	1119.57*	LOC100996288	0.08658
551.98	1109.97*	ARF3	-5.70600
545.68	1099.37*	NTSR1	-6.70716
543.65	1097.31*	FAM76A	0.16293
541.91	1095.82*	SULT2B1	1.39343
537.45	1088.89*	AHSG	0.58412
534.03	1084.06*	PITX3	0.12057
533.07	1084.14*	LINC01843	-0.49704
533.06	1086.12*	NKX2.1	-0.43686
533.03	1088.07*	MTURN	-0.17840
532.54	1089.08*	ZNF710.AS1	0.08927
527.17	1080.35*	ANGPTL4	0.30854

Table S3: Prognosis-related key genes screening by the Cox model and clinically-integrated RNA-Seq data

nloglik	AIC	gene symbol	regression coefficients
546.32	1096.65*	LINC01843	-0.453
542.39	1090.78*	ZNF710.AS1	0.0749
539.98	1087.95*	AHSG	0.445
536.72	1083.44*	NTSR1	-4.38
533.49	1078.97*	PITX3	-0.0414
530.32	1074.63*	LINC00908	0.957
527.57	1071.14*	STAP1	0.291
527.38	1072.77*	FCRL1	120
526.30	1072.61*	SULT2B1	3.22
525.15	1072.29*	MS4A1	-31.0
522.52	1069.05*	ARF3	-6.70
520.47	1066.94*	LOC100996288	0.0618
517.59	1063.18*	ANGPTL4	0.333

Table S4: Prognosis-related key genes screening by the RSF model and RNA-Seq data

nloglik	AIC	gene symbol	regression coefficients
565.65	1133.30*	LINC00908	-0.0268
559.83	1123.66*	CRCT1	0.1390
552.81	1111.61*	ARF3	-0.4191
549.67	1107.33*	ZNF710.AS1	-0.0848
548.97	1107.93*	BAIAP2L2	-0.2632
542.15	1096.30*	ANGPTL4	0.2133
540.87	1095.75*	MAFK	0.0785
540.66	1097.33*	C1orf106	0.5986
535.43	1088.86*	PITX3	-0.1456
534.48	1088.96*	KRT18	-0.0980
532.04	1086.08*	GABRA2	-0.3414
531.40	1086.80*	SLC2A1	0.4923
529.68	1085.36*	LOC100996732	0.3154
528.04	1084.07*	FAM117A	-0.0862
525.98	1081.96*	CNNM1	-2.4091

Table S5: Prognosis-related key genes screening by the RSF model and clinically-integrated RNA-Seq data

nloglik	AIC	gene symbol	regression coefficients
548.77	1101.54*	LINC00908	-0.156651
543.01	1092.02*	PITX3	-0.105483
539.34	1086.68*	GJB3	0.482751
537.21	1084.43*	CRCT1	0.540022
535.83	1083.65*	MELTF	0.243086
533.19	1080.39*	BAIAP2L2	-0.128905
530.44	1076.87*	RHOV	-8.397672
527.78	1073.55*	GABRA2	-1.585957
526.12	1072.24*	ARF3	-0.657799
524.48	1070.96*	TRIM7	-0.047925
522.88	1069.76*	KRT18	-0.005595
521.29	1068.58*	ZNF710.AS1	-0.513451
517.79	1063.57*	LOC105370802	-0.101560
517.26	1064.52*	LOC100996732	0.263032
517.09	1066.19*	SFTPB	1.604695
513.51	1061.01*	DKK1	-0.137363

Table S6: Parameter setting of the forward selection model

model	max.n.genes	n.iter
model 1[a]	100	35
model 2[b]	90	20
model 3[c]	95	35
model 4[d]	95	10

[a]model 1: the Cox model and RNA-Seq data;
[b]model 2: the Cox model and clinically-integrated RNA-Seq data;
[c]model 3: the RSF model and RNA-Seq data;
[d]model 4: the RSF model and clinically-integrated RNA-Seq data.

Table S7: Cox survival analysis of the GSE72094 cohort

Factor	Univariate		Multivariable	
	HR[a](95% CI[b])	*p*	HR(95% CI)	*p*
age(>70[c] vs <=70)	1.12 (0.77 to 1.62)	0.56	1.07 (0.73 to 1.56)	0.75
gender(female vs male)	0.65 (0.45 to 0.94)	0.026	0.54 (0.33 to 0.89)	0.02
stage	1.62 (1.36 to 1.94)	8.88e-08	1.73 (1.36 to 2.19)	5.61e-06
risk score (high vs low)	2.12 (1.44 to 3.12)	1.47e-04	0.91 (0.50 to 1.64)	0.75

[a]HR = hazard ratio;
[b]CI = confidence interval;
[c]70 = median age of patients with LUAD in the GSE72094 cohort.

Table S8: Cox survival analysis of the GSE11969 cohort

Factor	Univariate		Multivariable	
	HR[a](95% CI[b])	*p*	HR(95% CI)	*p*
age(>63[c] vs <=63)	1.61 (1.02 to 2.54)	0.04	1.73 (1.08 to 2.76)	0.02
gender(female vs male)	0.70 (0.43 to 1.17)	0.18	0.77 (0.45 to 1.31)	0.33
stage	1.99 (1.54 to 2.58)	1.86e-07	1.72 (1.05 to 2.80)	0.03
risk score (high vs low)	3.37 (2.06 to 5.49)	1.18e-06	1.48 (0.59 to 3.74)	0.40

[a]HR = hazard ratio;
[b]CI = confidence interval;
[c]63 = median age of patients with LUAD in the GSE11969 cohort.

Table S9: Survival analysis based on the GSE11969 cohort

model	HR[a] (95% CI[b])	*p*	C-index[c]
model 1[d]	1.89 (1.19-3.00)	7.49e-03	0.590
model 2[e]	3.60 (2.18-5.94)	5.69e-07	0.660
model 3[f]	1.89 (1.18-3.02)	8.39e-03	0.590
model 4[g]	3.87 (2.27-6.61)	6.81e-07	0.670

[a]HR = hazard ratio;
[b]CI = confidence interval;
[c]C-index = concordance index;
[d]model 1: the Cox model and RNA-Seq data;
[e]model 2: the Cox model and clinically-integrated RNA-Seq data;
[f]model 3: the RSF model and RNA-Seq data;
[g]model 4: the RSF model and clinically-integrated RNA-Seq data.
